# Reduced protocadherin17 expression in leukemia stem cells: the clinical and biological effect in acute myeloid leukemia

**DOI:** 10.1186/s12967-019-1851-1

**Published:** 2019-03-29

**Authors:** Zi-jun Xu, Ji-chun Ma, Jing-dong Zhou, Xiang-mei Wen, Dong-ming Yao, Wei Zhang, Run-bi Ji, De-hong Wu, Li-juan Tang, Zhao-qun Deng, Jun Qian, Jiang Lin

**Affiliations:** 1 Laboratory Center, Affiliated People’s Hospital of Jiangsu University, 8 Dianli Rd., Zhenjiang, 212002 Jiangsu People’s Republic of China; 2 Zhenjiang Clinical Research Center of Hematology, Zhenjiang, 212002 Jiangsu People’s Republic of China; 3 The Key Lab of Precision Diagnosis and Treatment in Hematologic Malignancies of Zhenjiang City, Zhenjiang, 212002 Jiangsu People’s Republic of China; 4 Department of Hematology, Affiliated People’s Hospital of Jiangsu University, 8 Dianli Rd., Zhenjiang, 212002 Jiangsu People’s Republic of China; 5 Department of Clinical Laboratory Medicine, Affiliated People’s Hospital of Jiangsu University, Zhenjiang, 212002 People’s Republic of China; 6 Department of Hematology, The Third People’s Hospital of Kunshan City, Kunshan, 215300 Jiangsu People’s Republic of China

**Keywords:** Protocadherin17, Acute myeloid leukemia, DNA methylation, Gene expression, Prognosis

## Abstract

**Background:**

Leukemia stem cell (LSC)-enriched genes have been shown to be highly prognostic in acute myeloid leukemia (AML). However, the prognostic value of tumor suppressor genes (TSGs) that are repressed early in LSC remains largely unknown.

**Methods:**

We compared the public available expression/methylation profiling data of LSCs with that of hematopoietic stem cells (HSCs), in order to identify potential tumor suppressor genes in LSC. The prognostic relevance of *PCDH17* was analyzed on a cohort of 173 AML patients from The Cancer Genome Atlas (TCGA), and further validated in three independent cohorts (n = 339).

**Results:**

We identified protocadherin17 (*PCDH17*) and demonstrated that it was significantly down-regulated and hypermethylated in LSCs compared with HSCs. Our analyses of primary AML patient samples also confirmed these deregulations. Clinically, low *PCDH17* expression was associated with female sex (*P* = 0.01), higher WBC (*P* < 0.0001), higher percentages of blasts in bone marrow (BM) and peripheral blood (PB) (*P* = 0.04 and < 0.001, respectively), presence of *FLT3*-internal tandem duplications (*P* = 0.002), mutated *NPM1* (*P* = 0.02), and wild-type *TP53* (*P* = 0.005). Moreover, low *PCDH17* expression predicted worse overall survival (OS) in four independent cohorts as well as in the molecularly defined subgroups of AML patients. In multivariable analyses, low *PCDH17* expression retained independent prognostic value for OS. Biologically, *PCDH17* expression-associated gene signatures were characterized by deregulations of EMT- and Wnt pathway-related genes.

**Conclusions:**

*PCDH17* gene was silenced by DNA methylation in AML. Low *PCDH17* expression is associated with distinct clinical and biological features and improves risk stratification in patients with AML.

**Electronic supplementary material:**

The online version of this article (10.1186/s12967-019-1851-1) contains supplementary material, which is available to authorized users.

## Background

Acute myeloid leukemia (AML) is a group of hematological malignancies marked by heterogeneity in its biological features and clinical outcomes. The biological heterogeneity mainly consists of cytogenetic [1], genetic [2, 3], epigenetic [4], and transcriptional diversities [5, 6]. These aberrations together contribute to the clinical heterogeneity of AML, which makes it difficult for risk stratification and targeted therapy of the disease. To date, karyotypic analysis remains as the mainstay of risk classification in AML, yet 40 to 50% of patients lack clonal chromosome aberrations [1]; molecular markers, especially recurrent somatic mutations, have shown their ability to subdivide this large group of patients [7]. As described by the latest WHO classification of AML [8], mutations in *NPM1* (without concomitant *FLT3*-ITD) and biallelic mutations of *CEBPA* in cytogenetically normal patients are associated with a favorable prognosis, whereas *RUNX1* mutations have been implicated in worse overall survival in AML patients. Many other mutations, however, are not included in current clinical practice, primarily due to inconsistent prognostic findings and a lack of validation in large cohorts. Moreover, there is considerable prognostic variability even in the genetically defined subclasses. Thus, novel biomarkers, like gene expression alterations, are needed to improve outcome prediction in the context of established prognostic markers in AML.

There is now ample evidence that AML is initiated and maintained by leukemia stem cells (LSCs) [9]; this subset of cells is deemed to mediate drug resistance and disease relapse of AML. Importantly, recent studies have generated LSC-specific gene expression signatures that are highly prognostic in multiple independent cohorts of AML patients [10–13], demonstrating the potential utility of testing LSC genes expression in clinical practice. Previously, it was found that haploinsufficiency of Dnmt1, a DNA methyltransferase, effectively impaired LSC function and lead to prolonged survival of MLL-AF9 induced leukemic models [14]. This leads us to speculate that some survival-related tumor suppressor genes (TSGs) may be repressed by DNA methylation in LSCs. Indeed, repression of stem cell-related TSGs has been shown to correlate with poor outcome and cancer progression in solid tumors [15]. As promoter methylation is a major mechanism in silencing TSGs expression, it would be of interest to identify aberrantly hypermethylated and repressed TSGs in LSCs, especially those genes that have profound biological and clinical implications.

Protocadherin17 (*PCDH17*), a member of the cadherin superfamily, is predominantly expressed in the nervous system and has its major roles in axon extension, synapse formation, and dendrite arborization [16]. The gene locates on chromosome 13q21, a region for which homozygous deletion often occurred in cancers [17]; this suggests *PCDH17* as a potential tumor suppressor beyond its role in neural development. In line with this, *PCDH17* gene is found transcriptionally silenced in various human cancers, due to mechanisms including deletion, mutation, and more often, promoter methylation [18, 19]. In esophageal squamous cell carcinoma (ESCC), *PCDH17* was preferentially silenced in poorly differentiated tumors and re-expression of *PCDH17* reduced proliferation and migration of ESCC cells [20]. Frequent epigenetic inactivation of *PCDH17* was also reported in gastric and colorectal cancers, in which *PCDH17* exerts its tumor suppressive activity by inducing apoptosis and autophagy [21]. Importantly, *PCDH17* received as much attention for its diagnostic and prognostic implications—as in urologic neoplasms, where *PCDH17* methylation was reported to be a highly sensitive and specific diagnostic marker [22]. Despite progress in understanding its tumor-suppressive functions and clinical relevance in solid tumors, the role of *PCDH17* in hematological malignancies remains largely unknown. Indeed, a recent finding in pediatric acute lymphoblastic leukemia has shown that *PCDH17* methylation was significantly associated with disease relapse and worse outcome [23], thereby suggesting a potential involvement of *PCDH17* in leukemogenesis.

In this study, we identified six potential tumor suppressors that are specifically repressed and hypermethylated in LSCs, including *PCDH17*. The clinical impact of aberrant *PCDH17* expression was further explored in the context of known molecular prognosticators in AML. Furthermore, to gain insights into molecular features of *PCDH17*-deregulated AML, we generated gene- and microRNA-expression signatures associated with changes in *PCDH17* expression levels, using RNA and microRNA sequencing data derived from the Cancer Genome Atlas (TCGA) AML project [3].

## Methods

### Patients and samples

To analyze the prognostic impact of *PCDH17* in AML patients, four independent cohorts with survival information were included in this study: (1) a discovery cohort including 200 de novo AML patients derived from the TCGA AML study [3]; (2) two validation cohorts of 242 cytogenetically normal AML patients described by Metzeler et al. [24]; (3) an independent (Chinese) cohort consisted of 184 AML patients enrolled from 2005 to 2016 and treated in the Affiliated People’s Hospital of Jiangsu University. Detailed patient characteristics and treatment of the three published cohorts were described in Additional file 1: Additional Methods. For the Chinese cohort, all patients received induction and consolidation chemotherapy as described previously [25]. Of the 184 patients studied, *PCDH17* expression data were obtained by real-time quantitative PCR (RT-qPCR) for 126 patients and 97 of them have survival data (OS only), while *PCDH17* methylation levels were detected by targeted bisulfite sequencing for 107 patients. To test the association between *PCDH17* expression and chemotherapy, we collected 20 paired BM samples from 10 patients both at diagnosis and during complete remission. Mutation and cytogenetic analyses were performed as previously described [25]. For comparison with normal controls, bone marrow samples from 66 healthy donors were collected. This study was approved by the Institutional Ethics Committee of the Affiliated People’s Hospital of Jiangsu University.

### Public datasets

We used eight public available AML datasets from TCGA and Gene Expression Omnibus in this study (see Additional file 1: Table S1 for details). Of these data sets, two of them consists of expression data for bulk primary AML samples (GSE12417 and the TCGA dataset), and four datasets contain both healthy and AML BM samples sorted by fluorescence-activated cell sorting (FACS) (GSE24006, GSE30029, GSE63270, and GSE63409). Profiling data of the HSC and LSC subpopulations extracted from GSE63270 to GSE63409 were used for discovering TSGs that are down-regulated and hypermethylated in LSCs. For GSE24006 and GSE30029—both of which were available for expression data from CD^34+^ and CD^34−^ samples—only data of the CD^34+^ populations were utilized, as the majority of LSCs were found to reside in this compartment [11]. We also used a gene-expression dataset of normal hematopoietic cells (GSE42519) to assess the expression pattern of *PCDH17* at various stages of normal hematopoiesis. In addition, two manually-aggregated datasets from BloodSpot (http://servers.binf.ku.dk/bloodspot/) were used, that is, HemaExplorer, which contains samples of normal hematopoietic cell lineages from four studies (GSE17054, GSE19599, GSE11864, and E-MEXP-1242), and BloodPool, a large dataset containing bulk AML samples and normal hematopoietic cell samples assembled from 6 independent studies (GSE13159, GSE15434, GSE61804, GSE14468, TCGA, and GSE42519). For each microarray dataset, the probe set with the highest average expression was selected to represent *PCDH17* expression level. See Additional file 1: Additional Methods for detailed information and analyses of these data sets.

### Real-time quantitative reverse transcriptase-PCR (RT-qPCR)

Total RNA was extracted using TRIzol reagent (Invitrogen, Carlsbad, CA, USA) and reverse transcribed into cDNA as described previously [26]. Real-time quantitative PCR (RT-qPCR) was performed using AceQ qPCR SYBR Green Master Mix (Vazyme Biotech Co., Piscataway, NJ, USA) on a 7500 Thermo cycler (Applied Biosystems, CA). Reaction conditions and PCR cycling were conducted as previously described [26], adjusting only for optimized primer annealing temperatures (60 °C). PCR primers were designed using Primer Premier 6 (Premier Biosoft, Palo Alto, CA, USA), and the primer sequences were listed in Additional file 1: Table S2. The relative quantification was calculated using the ΔΔCT method and normalized to the *ABL1* housekeeping gene.

### Targeted bisulfite sequencing

Genomic DNA was extracted from bone marrow cells with Puregene Blood Core Kit B (QIAGEN Sciences, MD, USA). The DNA Concentration and purity were assessed using Nanodrop 2000 (Thermo Fisher Scientific, MA, USA), then DNA was subjected to bisulfite modification by EZ DNA Methylation-Gold Kit (ZYMO, CA, USA), following the manufacturer’s instructions. Primers were designed using primer3 (http://primer3.ut.ee/) based on the bisulfite converted DNA. To prepare for high-throughput DNA sequencing, the target sequence was subjected to multiplexed PCR amplification followed by index PCR, the PCR products were further purified to generate sequencing library. Paired-end sequencing (2 × 150 bp) was performed on the Illumina MiSeq platform (Illumina, San Diego, CA, USA). After quality assessment and filtering, paired reads generated from the Illumina MiSeq platform were merged using FLASH (Fast length adjustment of short reads) [27], the merged reads were then aligned (mapped) to the human GRCh37/hg19 genome assembly (in silico bisulfite-converted human reference genome [GRCh37]) using blast + [28]. CpG methylation values were calculated as methylated reads counts/total read counts of every CG point. Principal component analysis (PCA) was conducted using the “FactoMineR” package in R, to determine similarities of methylation levels between the two groups; two principal components (PC1 and PC2, representing the largest two variances) were plotted using “fviz_pca_ind” function from “factoextra” R package.

### Cell culture, 5-aza-dC treatment and real-time methylation-specific polymerase chain reaction (RQ-MSP)

Human leukemia cell line HL60 (American Type Culture Collection, Manassas, VA, USA) was cultured in RPMI 1640 medium plus 10% fetal calf serum (ExCell Bio, Shanghai, China). Cells were incubated in a humidified atmosphere containing 5% CO_2_ at 37 °C. The cells were placed at a density of 5 × 10^5^ cells/ml in 5 mL medium 24 h before treatment. Graded doses (0 μM, 2 μM, and 4 μM) of 5-aza-2′-deoxycytidine (5-aza-dC) (Sigma-Aldrich, Steinheim, USA) were then added to the medium. After 3 days of treatment, the cells were harvested. Extractions of RNA and DNA were performed as described above. To assess the changes in methylation level, real-time methylation-specific polymerase chain reaction (RQ-MSP) was employed using primers listed in Additional file 1: Table S2. PCR conditions were as described previously [26], except that the annealing temperature for methylation primers was 59 °C.

### Statistical analysis and bioinformatics

Overall survival (OS) was defined as the time from the date of diagnosis to death due to any cause. Disease-free survival (DFS) was defined as the period after primary treatment during which there are no signs and symptoms of the disease. For the datasets analyzed, patients were stratified as high *PCDH17* expression and low *PCDH17* expression, according to the median expression value. Survival probabilities of the two groups were estimated by the Kaplan–Meier method and compared by log-rank test. Those variables with log-rank p-values less than or equal to 0.20 were further included in a multivariate Cox regression model. All survival analyses were performed using the “survival” R package, with the survival curves drawn by R package “survminer”.

To test for the significance of the differences across groups, Chi square tests or Fisher’s exact test was used for categorical data. For continuous data, a nonparametric test such as the Wilcoxon rank-sum test was used. Spearman correlation analysis was performed to determine the association between two continuous variables. All basic statistical analyses were performed using the base functions in R version 3.4 (https://www.r-project.org). Differential gene expression analysis for RNA/microRNA sequencing data was calculated using the raw read counts with the R/Bioconductor package “edgeR”, while package “limma” was used for gene-expression and DNA-methylation microarray data, all analyses were controlled for the false discovery rate (FDR) by the Benjamini–Hochberg procedure. Gene set enrichment analysis (GSEA) was performed on the TCGA dataset using GSEA v3.0 software (http://www.broad.mit.edu/gsea). All plots and graphs were generated using the R package “ggplot2” unless otherwise stated. For details of bioinformatics see Additional file 1: Additional Methods.

## Results

### PCDH17 is significantly down-regulated in AML and dynamic expressed during normal hematopoiesis

**Fig. 1:**
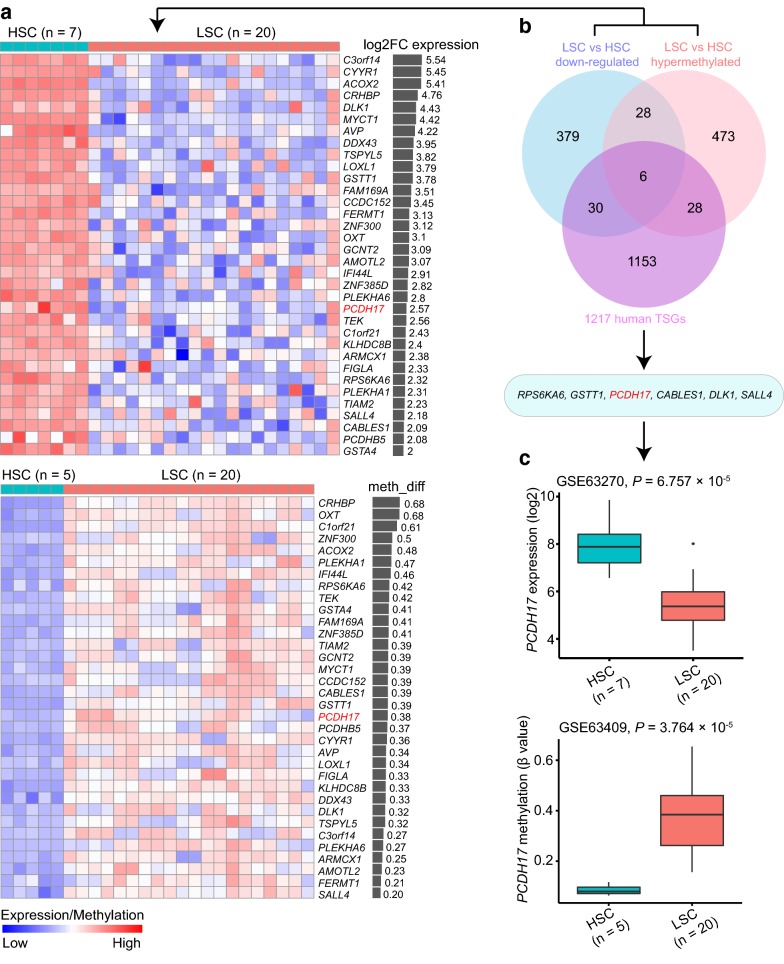
Identification of potential tumor suppressor genes that are repressed early in LSC. **a** Heatmaps showing down-regulated genes (upper panel) and hypermethylated genes (lower panel) in LSCs versus HSCs, from GEO: GSE63270 and GEO: GSE63409, respectively. Log2 fold changes of gene expression (log2 FC expression, upper panel) and β-value differences in DNA methylation (meth_diff, lower panel) are displayed as bar graphs on the right. The heatmaps only represent the 34 genes that are simultaneously down-regulated and hypermethylated in LSCs versus HSCs. For LSC, n = 20 represents 20 LSC fractions from 10 patients. **b** Venn diagram showing the overlap of three gene sets including: 443 down-regulated genes (FDR < 0.05, log2 FC > 2) in LSCs versus HSCs, 535 hypermethylated genes (FDR < 0.05, methylation difference > 20%) in LSCs versus HSCs, and 1217 TSGs from the tumor suppressor gene database. The overlapping region (6) represents the finally screened tumor suppressor genes. **c** Boxplots showing gene expression (upper panel) and DNA methylation (lower panel) changes of *PCDH17* in LSCs versus HSCs, from GEO: GSE63270 and GEO: GSE63409, respectively. The *P* values calculated with a Wilcoxon rank-sum test are shown

To identify candidate tumor suppressor genes in LSCs, the dataset GSE63270 (gene-expression data, n = 104) and GSE63409 (DNA-methylation data, n = 74) were utilized [12]. The two datasets were profiled from FACS sorted BM samples from 15 AML patients and 5 healthy controls. For our purpose, we only extracted data concerning the LSC fractions (n = 20 for both datasets) and the HSC subpopulations (GSE63270, n = 7; GSE63409, n = 5), in which the LSC phenotype was determined by their surface immunophenotype and leukemic engraftment ability [12]. Differential expression/methylation analyses were then performed between these two populations. Our analyses yielded 443 significantly down-regulated genes/probes (FDR < 0.05, log2 FC > 2, Additional file 2) in LSCs versus HSCs. For DNA methylation data, 863 hypermethylated CpG sites located in the promoter regions (FDR < 0.05, methylation difference > 20%, Additional file 3) were identified, corresponding to 535 unique genes. By intersecting the two gene lists, we identified that 34 genes were simultaneously down-regulated and hypermethylated in LSCs compared to HSCs (Fig. 1a). The 34 genes were further checked in the tumor suppressor gene database including 1217 TSGs [29]. Six genes (*RPS6KA6*, *GSTT1*, *PCDH17*, *CABLES1*, *DLK1*, and *SALL4*) were at last screened (The overlap of three gene sets was shown as a Venn diagram in Fig. 1b). Among them, *PCDH17* has been reported as a negative regulator of the Wnt/β-catenin pathway [30], and since our group has already established the prognostic significance of several Wnt antagonists in AML [31–33], subsequent studies focused on the clinical impact of *PCDH17* gene.

**Fig. 2:**
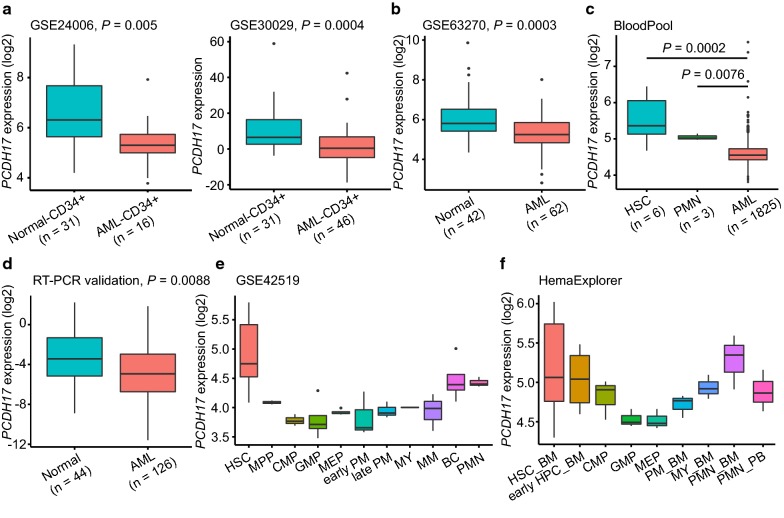
*PCDH17* expression in AML and normal hematopoiesis. **a**, **b** Box plots illustrating the validation of *PCDH17* expression changes in AML in three independent datasets. **a** Datasets using CD34 + cells from normal controls and AML patients, as indicated. **b** Dataset using whole bone marrow cells from normal controls and AML patients, as indicated. **c** Box plots showing that *PCDH17* expression is significantly lower in patients with AML in comparison to HSC and PMN from healthy donors, using a large aggregated data set (BloodPool) from the BloodSpot website (http://servers.binf.ku.dk/bloodspot/). **d** Box plot comparing *PCDH17* expression levels in normal controls and AML as assessed by RT-qPCR. Comparisons were made by the Wilcoxon rank-sum test. **e** Box plot showing the expression pattern of *PCDH17* during differentiation of the major hematopoietic lineages using published dataset (GSE42519). **f** Box plot showing the expression pattern of *PCDH17* during differentiation of the major hematopoietic lineages using a merged data set (HemaExplorer) from the BloodSpot website (http://servers.binf.ku.dk/bloodspot/). *HSC* hematopoietic stem cell, *MPP* multipotent progenitor, *CMP* common myeloid progenitor, *GMP* granulocyte-monocyte progenitor, *MEP* megakaryocyte-erythrocyte progenitor, *PM* promyelocyte, *MY* myelocyte, *MM* metamyelocytes, *BC* band cell, *PMN* polymorphonuclear cells, *HPC* hematopoietic progenitor cells, *BM* bone marrow, *PB* peripheral blood

The expression and DNA methylation changes of *PCDH17* were displayed as boxplots regarding the two discovery datasets (Fig. 1c). Further comparing *PCDH17* expression between CD^34+^ AML and CD^34+^ normal samples in two independent datasets (GSE24006 and GSE30029), we also detected a significant reduction of *PCDH17* expression in CD^34+^ AML cells (GSE24006, n = 16; GSE30029, n = 46), as compared with CD^34+^ normal cells (n = 31 for both datasets) (Fig. 2a). A similar result was observed when the total AML—(n = 62) and normal—(n = 42) mononuclear cell (MNC) fractions of the GSE63270 dataset were analyzed (Fig. 2b). Next, using the BloodPool dataset from BloodSpot, we show that *PCDH17* expression is significantly lower in patients with AML (n = 1825) in comparison to HSC (n = 6) and polymorphonuclear cell (PMN) (n = 3) from healthy donors (Fig. 2c). This repression was further verified through RT-qPCR on 126 bulk primary AML and 44 normal BM samples (Fig. 2d). Together, these results demonstrate that transcriptional silencing of *PCDH17* is a common feature in AML patients.

To extend these findings and determine the expression pattern of *PCDH17* during normal hematopoiesis, the level of *PCDH17* transcripts was analyzed using a published data (GSE42519), in which gene expression profiling was performed with samples from normal hematopoiesis [34]. We found *PCDH17* transcripts was highest in BM HSCs, with a sharp reduction in committed progenitors (common myeloid progenitor, granulo-monocyte progenitor, megakaryocyte-erythroid progenitor), the expression remained low and then gradually increased during myeloid maturation (Fig. 2e). The dynamic expression pattern of *PCDH17* during normal hematopoiesis was further confirmed in a merged data set (HemaExplorer) from BloodSpot (Fig. 2f). This dynamic regulation of *PCDH17* transcripts suggests a potential role of *PCDH17* in HSC biology.

### Repression of PCDH17 is mediated by promoter methylation in AML

**Fig. 3:**
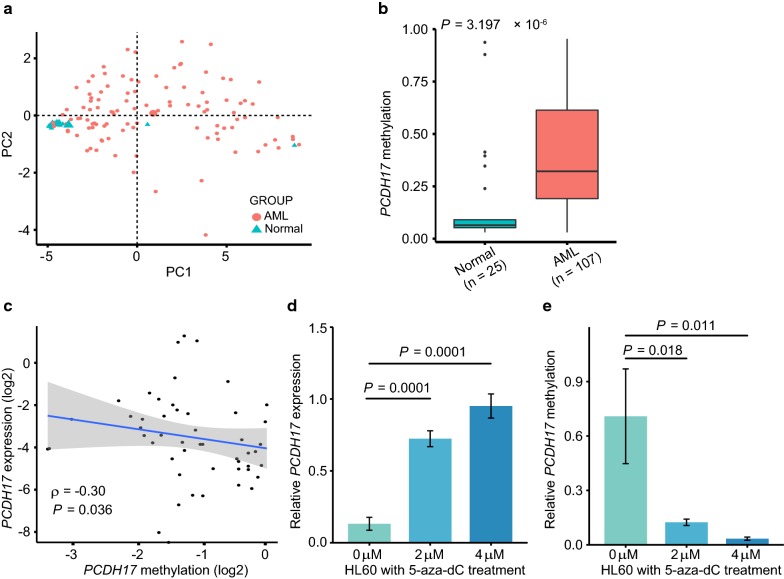
Methylation-mediated silencing of *PCDH17* expression in AML. **a** Principal component analysis (PCA) of *PCDH17* methylation levels determined by targeted bisulfite sequencing. Methylation data of the measured 20 CpG sites across 132 samples (107 AML and 25 normal BM- derived MNCs) is provided in Additional file 4. **b** Box plot showing average *PCDH17* methylation differences in normal controls and AML patients, calculated using the Wilcoxon rank-sum test. **c** Scatterplot showing the correlation between *PCDH17* methylation and *PCDH17* expression in AML patients. Spearman’s correlation coefficient (ρ) and *P* value are indicated. **d**, **e** *PCDH17* expression (**d**) and methylation (**e**) in HL60 cell line before and after 5-aza-dC treatment. The data are shown as mean ± S.D. of triplicate analyses. Statistical significances were determined using Student’s t-tests

To assess the methylation pattern of *PCDH17* in primary AML samples and to see whether its repression is mediated by methylation, we used targeted bisulfite sequencing to measure the methylation levels of 20 CpG sites in the first exon of *PCDH17* (chr13:58,206,837-58207013). Detailed methylation data of this sequenced region was provided in Additional file 4. PCA analysis of our methylation data showed a clear discrimination between AML and normal BM-derived MNCs (Fig. 3a). The β values of the 20 CpG sites were then averaged as the DNA methylation level of *PCDH17* gene, which was significantly higher in AML patients (n = 107; median, 0.3219; range, 0.0303-0.9542; *P* < 0.0001) compared with the healthy controls (n = 25; median, 0.0649; range, 0.0302-0.9372) (Fig. 3b).

Furthermore, as shown in Fig. 3c, *PCDH17* methylation levels were negatively correlated with mRNA levels (ρ = − 0.30, *P* = 0.036). To determine whether aberrant *PCDH17* methylation could be reverted in vitro, we treated HL60 cells with demethylation reagent 5-aza-dC. Our results showed that 5-aza-dC treatment significantly up-regulated *PCDH17* expression (Fig. 3d); this was supported by the MSP analyses, which also showed a change in the methylation status of the *PCDH17* promoter (Fig. 3e). In summary, these results suggested *PCDH17* expression is inactivated, at least in part, by its promoter methylation in AML.

### PCDH17 expression is associated with clinical characteristics and molecular markers

For the discovery cohort, the association of the clinical and genetic characteristics with *PCDH17* expression was summarized in Table 1. At diagnosis, patients with low *PCDH17* expression were more likely to be females (*P* = 0.01), with higher white blood cell (WBC) counts (*P* < 0.0001), higher percentage of blasts in BM and PB (*P* = 0.04 and < 0.001, respectively). There were no other significant differences in presenting clinical characteristics between these two groups including age (*P* = 0.41) and karyotype classification (*P* = 0.35).

**Table 1:** Patient characteristics with respect to *PCDH17* expression

Patient characteristics	AML (TCGA dataset)		
	High *PCDH17* (n = 86)	Low *PCDH17* (n = 87)	*P*
Age, years			0.41
Median	59	57	
Range	18–88	21–82	
Sex (male/female)	54/32	38/49	0.01
WBC count, × 10^9^/L			< 0.0001
Median	9.3	34	
Range	0.4–116.2	0.8–297.4	
BM blasts, %			0.04
Median	68.5	75	
Range	30–100	32–100	
PB blasts, %			< 0.001
Median	18	49	
Range	0–97	0–98	
Karyotype			0.343
Favorable	16	16	
Intermediate	46	55	
Adverse	23	14	
Unknown	1	2	
*FLT3*-*ITD*			0.002
Present	6	21	
Absent	80	66	
*NPM1*			0.02
Mutated	17	31	
Wild-type	69	56	
*CEBPA*			1
Single mutated	4	4	
Double mutated	2	3	
Wild-type	80	80	
*IDH1*			0.28
Mutated	10	6	
Wild-type	76	81	
*IDH2*			0.82
Mutated	8	9	
Wild-type	78	78	
*RUNX1*			0.769
Mutated	8	7	
Wild-type	78	80	
*DNMT3A*			0.755
Mutated	20	22	
Wild-type	66	65	
*TP53*			0.005
Mutated	12	2	
Wild-type	74	85	
*ERG* expression^a^			0.08
High	37	49	
Low	49	38	
*BAALC* expression^a^			0.494
High	45	41	
Low	41	46	
*MN1* expression^a^			0.704
High	44	42	
Low	42	45	
*WT1* expression^a^			0.323
High	40	47	
Low	46	40	

*AML* acute myeloid leukemia, *TCGA* The Cancer Genome Atlas, *WBC* white blood cells, *BM* bone marrow, *PB* peripheral blood, *ITD* internal tandem duplication

^a^The median expression value was used as a cut point

With respect to known prognostic markers, patients with low *PCDH17* expression more often had *FLT3*-ITD (*P* = 0.002) and *NPM1* mutations (*P* = 0.02) and less often had *TP53* mutations (*P* = 0.005) than patients with high *PCDH17* expression. These associations were confirmed by comparing *PCDH17* expression levels as stratified by mutation status of the three genes (Additional file 1: Figure S1a–c). No significant differences were observed between low- and high- *PCDH17* expression patients regarding other molecular alternations, although low expressers had a trend toward more often having high expression of *ERG* (*P* = 0.08).

### Reduced PCDH17 expression predicts inferior survival in four independent cohorts

**Fig. 4:**
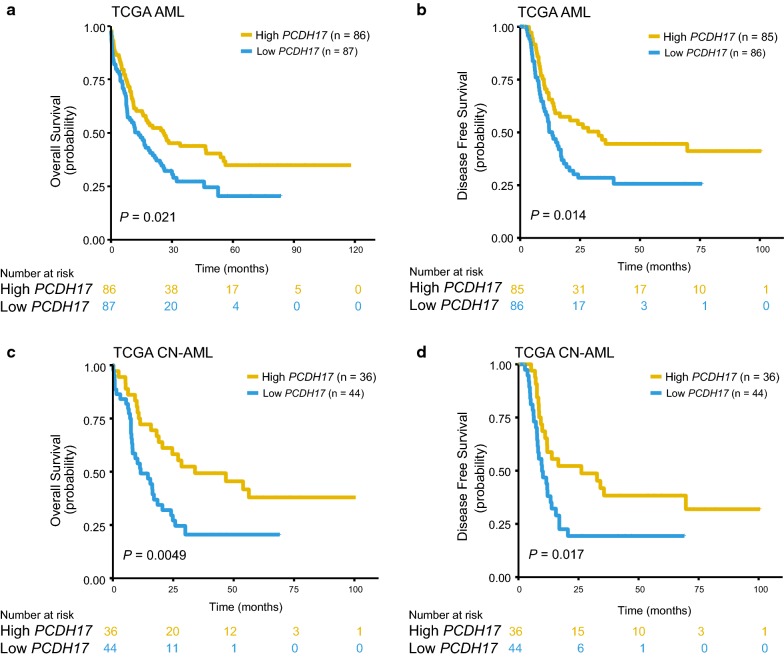
Prognostic significance of *PCDH17* in the discovery (TCGA) cohort. **a**, **b** The association of *PCDH17* expression levels with OS (**a**) and DFS (**b**) for the whole TCGA cohort. **c**, **d** The association of *PCDH17* expression levels with OS (**c**) and DFS (**d**) in the TCGA CN-AML patients. Patients were separated into high and low *PCDH17* group based on the median expression levels of *PCDH17*

**Fig. 5:**
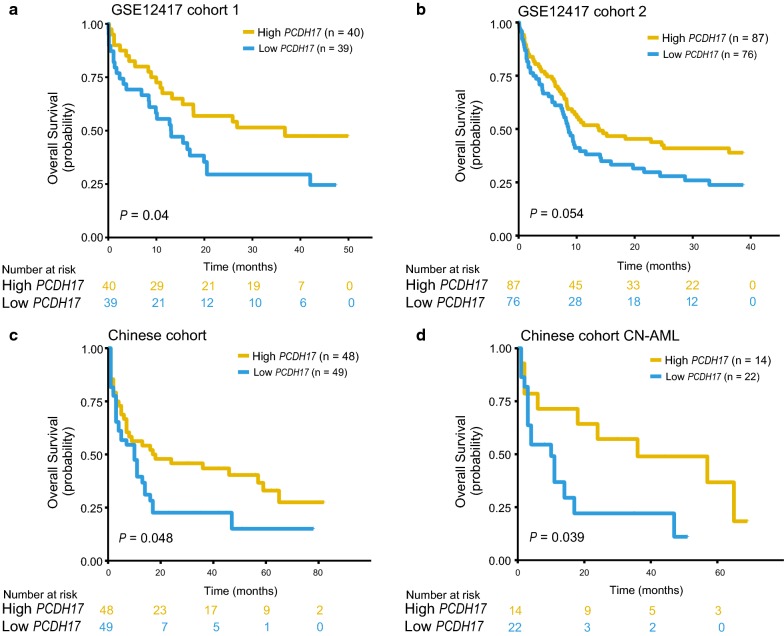
Validation of the prognostic value of *PCDH17* in three independent AML patient cohorts. **a**, **b** Kaplan–Meier analysis of overall survival (OS) in two CN-AML cohorts: GSE12417 cohort 1 (n = 79) (**a**) and GSE12417 cohort 2 (n = 163) (**b**). **c**, **d** Kaplan–Meier estimate for OS of the Chinese cohort in the whole (**c**) as well as in the CN-AML (**d**) patients. Patients were separated into high and low *PCDH17* group based on the median expression levels of *PCDH17*

To test the prognostic relevance of deregulated *PCDH17* expression, we investigated the correlation between *PCDH17* expression and clinical outcomes in four independent AML cohorts, all cohorts were dichotomized according to the median expression value of *PCDH17*. In the TCGA AML cohort (n = 173), patients with low *PCDH17* expression had significantly reduced OS (*P* = 0.021, Fig. 4a) and DFS (*P* = 0.014, Fig. 4b). This survival difference was also found for the subset of cytogenetically normal (CN)-AML patients (n = 80) (OS, *P* = 0.0049, Fig. 4c; DFS, *P* = 0.017, Fig. 4d). The prognostic value for OS was further validated in two CN-AML cohorts from GSE12417 (cohort1, n = 79, *P* = 0.04, Fig. 5a; cohort 2, n = 163, *P* = 0.054, Fig. 5b). When applied to the RT-qPCR data in our cohort, low *PCDH17* expression remained an adverse indicator for OS both in the whole cohort (n = 97) (*P* = 0.048, Fig. 5c) and in the CN-AML subsets (n = 36) (*P* = 0.039, Fig. 5d).

### Prognostic value of PCDH17 expression in the context of other risk factors in AML

To assess whether the prognostic impact of *PCDH17* expression is independent from knowing clinical factors, especially recurrent mutations found in AML, a univariate analysis was first conducted for the TCGA cohort (Detailed results were shown in Additional file 1: Table S3), variables considered significant (*P* ≤ 0.2) were further included in the multivariate Cox proportional hazard model. As shown in Table 2, low *PCDH17* expression maintained significant independent prognostic value with respect to OS (HR = 2.04; 95% CI, 1.37-3.04; *P* < 0.001), after adjustment for age group (*P* < 0.0001), cytogenetic risk group (*P* = 0.001), *TP53* mutation status (*P* = 0.004), *DNMT3A* mutation status (*P* = 0.03), and *RUNX1* mutation status (*P* = 0.08). The independent predictive value of *PCDH17* was also true for DFS (HR = 1.89; 95% CI 1.19–2.98; *P* = 0.007). In CN-AML patients, *PCDH17* down-regulation was still an independent poor prognostic factor for OS (HR = 2.02; 95% CI 1.13–3.62; *P* = 0.02) and a borderline poor prognostic factor for DFS (HR = 1.79; 95% CI 0.93–3.44; *P* = 0.08). Importantly, the independent prognostic value of *PCDH17* for OS was also validated in the Chinese cohort (Table 3).

**Table 2:** Multivariate analysis of *PCDH17* expression for overall survival and disease-free survival in the TCGA cohort

Variables	Overall survival		Disease-free survival	
	Hazard ratio (95% CI)	*P*	Hazard ratio (95% CI)	*P*
Full cohort	(n = 173)		(n = 171)	
*PCDH17* ^a^	2.04 (1.37–3.04)	< 0.001	1.89 (1.19–2.98)	0.007
Age^b^	2.90 (1.94–4.34)	< 0.0001	1.52 (0.96–2.43)	0.08
WBC count^c^	–	–	2.14 (1.36–3.39)	0.001
Cytogenetic risk^d^	1.84 (1.28–2.65)	0.001	1.39 (0.95–2.04)	0.09
*TP53* ^e^	2.76 (1.37–5.55)	0.004	2.64 (0.93–7.47)	0.07
*DNMT3A* ^e^	1.61 (1.05–2.48)	0.03	1.41 (0.85–2.33)	0.18
*RUNX1* ^e^	1.75 (0.94–3.26)	0.08	–	–

CN-AML	(n = 80)		(n = 80)	
*PCDH17* ^a^	2.02 (1.13–3.62)	0.02	1.79 (0.93–3.44)	0.08
Age^b^	2.38 (1.35–4.19)	0.003	2.19 (1.10–4.36)	0.03
WBC count^c^	1.42 (0.81–2.48)	0.22	1.65 (0.89–3.04)	0.11
*FLT3*-*ITD*^f^	–	–	1.34 (0.60–2.99)	0.47
*TP53* ^e^	–	–	4.09 (0.46–36.02)	0.20
*DNMT3A* ^e^	1.99 (1.13–3.52)	0.02	2.15 (1.15–4.03)	0.02
*IDH1* ^e^	0.61 (0.21–1.78)	0.36	0.68 (0.22–2.08)	0.49

Hazard Ratio > 1 or Hazard Ratio < 1 indicate a higher or lower risk. Only variables with a univariable P ≤ 0.20 were included in the multivariable models. See Additional file 1: Table S3 for a full list of variables evaluated in univariable analysis

*TCGA* The Cancer Genome Atlas, *CI* confidence interval, *WBC* white blood cells, *CN-AML* cytogenetically normal AML, *ITD* internal tandem duplication

^a^Low vs high expression

^b^ > 60 vs ≤ 60 years

^c^ ≥ 30 vs < 30 × 10^9^/L

^d^Adverse vs intermediate vs favorable

^e^Mutated vs wild type

^f^Present vs absent

**Table 3:** Univariate and multivariate analysis of *PCDH17* expression for overall survival in the Chinese cohort

Variables	Univariate analysis		Multivariate analysis	
	Hazard Ratio (95% CI)	*P*	Hazard Ratio (95% CI)	*P*
Full cohort	(n = 97)		(n = 97)	
*PCDH17* ^a^	1.65 (1–2.72)	0.05	2.01 (1.19–2.98)	0.01
Age^b^	3.07 (1.87–5.04)	< 0.0001	2.10 (1.18–3.61)	0.02
WBC count^c^	3.14 (1.92–5.14)	< 0.0001	2.06 (1.16–3.64)	0.01
Cytogenetic risk^d^	2.04 (1.49–2.78)	< 0.0001	1.60 (1.05–2.43)	0.03
*FLT3*-*ITD*^e^	0.80 (0.36–1.77)	0.58	–	–
*NPM1* ^f^	0.97 (0.39–2.43)	0.95	–	–
*CEBPA* ^f^	1.18 (0.53–2.62)	0.69	–	–
*DNMT3A* ^f^	1.66 (0.71–3.89)	0.24	–	–
*IDH2* ^f^	6.13 (0.8–47)	0.08	5.35 (0.63–45.16)	0.12
*NRAS* ^f^	1.18 (0.37–3.81)	0.78	–	–
*KRAS* ^f^	8.80 (2.53–30.55)	< 0.001	5.16 (1.13–23.59)	0.03
*U2AF1* ^f^	14.43 (1.81–115.4)	0.01	4.99 (0.37–67.94)	0.23
*SRSF2* ^f^	3.28 (0.78–13.85)	0.11	0.95 (0.12–7.46)	0.96
*SETBP1* ^f^	0.82 (0.11–5.96)	0.85	–	–
*KIT* ^f^	0.64 (0.16–2.64)	0.54	–	–

CN-AML	(n = 36)		(n = 36)	
*PCDH17* ^a^	2.58 (1.02–6.52)	0.04	2.95 (1.04–8.32)	0.04
Age^b^	1.39 (0.61–3.14)	0.43	–	–
WBC count^c^	3.05 (1.34–6.93)	0.008	3.26 (1.35–7.87)	0.009
*FLT3*-*ITD*^e^	0.48 (0.14–1.63)	0.24	–	–
*NPM1* ^f^	0.50 (0.15–1.69)	0.27	–	–
*CEBPA* ^f^	1.19 (0.40–3.50)	0.76	–	–
*DNMT3A* ^f^	1.25 (0.42–3.68)	0.69	–	–
*IDH2* ^f^	6.37 (0.76–53.48)	0.09	13.30 (1.23–143.32)	0.03
*NRAS* ^f^	3.55 (0.45–27.94)	0.23	–	–
*KRAS* ^f^	6.46 (1.37–30.46)	0.02	9.17 (1.72–48.86)	0.009
*SETBP1* ^f^	6.37 (0.76–53.48)	0.09	4.52 (0.48–42.10)	0.19

Hazard ratio > 1 or Hazard ratio < 1 indicate a higher or lower risk. Only variables with a univariable P ≤ 0.20 were included in the multivariable models. Mutations such as *IDH1* and *TP53* were either not analyzed or detected in our cohort, thus they were not included in this analysis

*CI* confidence interval, *WBC* white blood cells, *CN-AML* cytogenetically normal AML, *ITD* internal tandem duplication

^a^Low vs high expression

^b^ > 60 vs ≤ 60 years

^c^ ≥ 30 vs < 30 × 10^9^/L

^d^Adverse vs intermediate vs favorable

^e^Present vs absent

^f^Mutated vs wild type

**Fig. 6:**
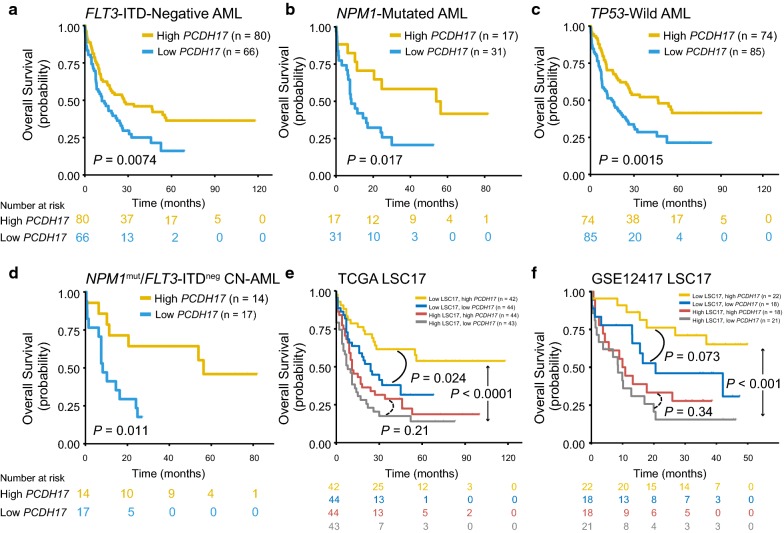
Reduced *PCDH17* expression predicts a worse outcome in the molecularly defined subgroups of AML patients. **a**–**d** Kaplan–Meier curves of OS stratified by *PCDH17* in the genetically defined subgroups of AML patients, as indicated. Low *PCDH17* expression is associated with poor outcome in all subgroups. **e**, **f** Kaplan–Meier curves of OS in the TCGA (**e**) and the GSE12417 cohort 1 (**f**) as stratified by the LSC17 signature. The low- and high-risk patients were further dichotomized by *PCDH17* expression status. *PCDH17* expression status could further stratify survival in the subgroup with low LSC17 score. Patients were separated into high and low *PCDH17* group based on the median expression levels of *PCDH17*

The strong prognostic significance of *PCDH17* status led us to hypothesize that it may provide additional prognostic information in the molecularly defined subgroups of AML patients. Indeed, exploratory subgroup analyses showed that the dichotomous stratification of *PCDH17* expression was still able to discriminate between shorter and longer OS in the *FLT3*-ITD absent, *NPM1* mutated, and *TP53* wild-type patients (*P* = 0.0074, 0.017, and 0.0015, respectively) (Fig. 6a–c), despite the aforementioned interaction among these alterations. Moreover, *PCDH17* status identified a poor-prognostic subset of patients within the *FLT3*-ITD^negative^/*NPM1*^mutated^ low-risk CN-AML group (*P* = 0.011, Fig. 6d), although this analysis is limited by a relatively small number of patients (n = 31). For DFS, however, we found that reduced *PCDH17* only had significant prognostic value in the *FLT3*-ITD absent and *TP53* wild-type subsets (Additional file 1: Figure S2).

A recently published risk score (LSC17) [13], generated using 17 LSC-specific genes, has proved to be a strong prognostic indicator independent from traditional prognosticators and other reported LSC signatures. We recalculated the LSC17 score (see Additional file 1 for code details) using the TCGA dataset and the GSE12417 dataset (cohort 1) and found it could accurately discriminate between shorter and longer OS in both cohorts (*P* < 0.0001 and *P* < 0.001, respectively) (Fig. 6e, f). When applied *PCDH17* status to the subsets of patients stratified by the LSC17 score, we found *PCDH17* expression further dichotomizes survival within the low-risk group (*P* = 0.024 for the TCGA cohort and *P* = 0.073 for the GSE12417 cohort) (Fig. 6e, f). This suggests that *PCDH17* could improve risk stratification in patients with a low LSC17 score.

### Association between PCDH17 expression and treatment response

**Fig. 7:**
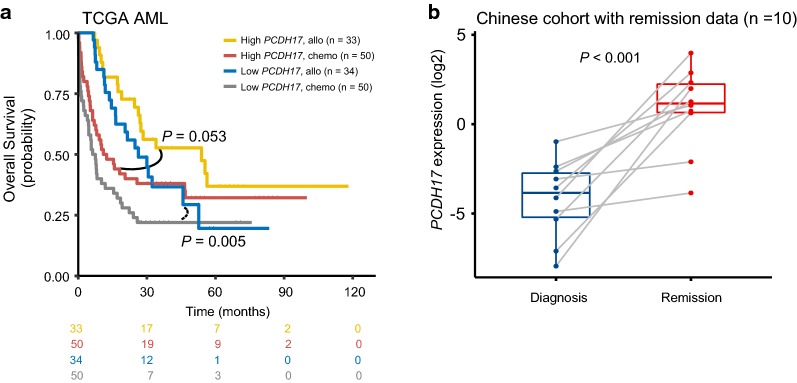
*PCDH17* expression may help predict treatment response in AML patients. **a** Kaplan–Meier curves of OS for patients with high or low *PCDH17* expression as stratified by treatment options (allo versus chemo) in the TCGA cohort. Allo, allogeneic transplantation; chemo, chemotherapy. **b** *PCDH17* expression changes in 10 follow-up patients with paired BM-MNC samples from diagnosis to remission in the Chinese cohort. Each grey line represents one patient. *PCDH17* expression was up-regulated in all patients after remission. The *P* value was calculated with a Wilcoxon rank-sum test

In the discovery cohort, 67 patients received allogeneic stem-cell transplantation (allo-HSCT); this therapy, though effective, is generally reserved for high-risk patients because of significant treatment-related mortality [35]. Thus, we asked whether patients with low *PCDH17* expression would benefit from allo-HSCT. We separately analyzed the prognostic impact of allo-HSCT in patients with low and high *PCDH17* expression. Results show that the benefit from allo-HSCT was more significant in patients with low *PCDH17* expression (*P* = 0.005), as compared with those with high expression (*P* = 0.053) (Fig. 7a). This suggests that patients with low *PCDH17* expression would potentially be candidates for allo-HSCT.

To further investigate *PCDH17* expression changes in the surveillance of AML, we assessed its expression in 10 patients with paired BM-MNC samples from diagnosis to complete remission (CR). Our data demonstrated that *PCDH17* expression was significantly increased in CR after induction therapy (*P* < 0.001, Fig. 7b).

### Distinct gene- and microRNA-expression signatures associated with PCDH17 expression in AML

**Fig. 8:**
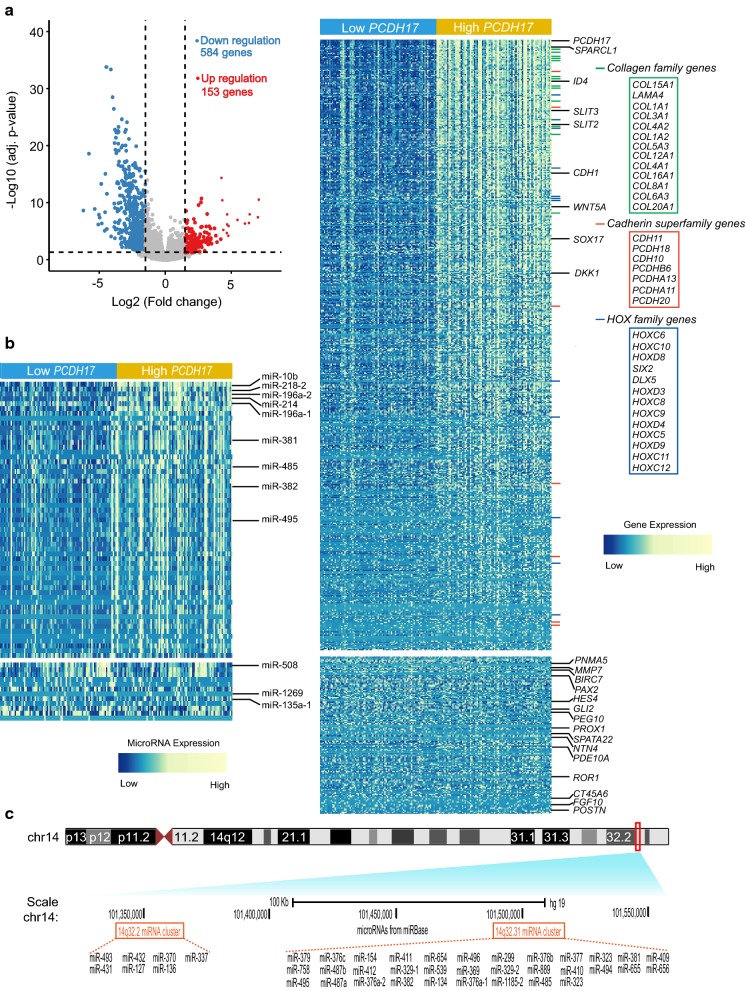
Gene/microRNA signatures associated with *PCDH17* expression. **a** Left: volcano plot showing gene expression differences between patients with low and high *PCDH17* expression. Significantly down-regulated genes (blue points) and up-regulated genes (red points) in patients with low *PCDH17* expression are indicated. Right: heatmap showing the gene expression signature associated with *PCDH17* expression (FDR < 0.05, |log2 FC| > 1.5). Up-regulated and down-regulated genes mentioned in the text are labeled on the rows or in the boxes. **b** Heatmap showing the microRNA expression signature associated with *PCDH17* expression (FDR < 0.05, |log2 FC| > 1.5). Up-regulated and down-regulated microRNAs mentioned in the text are indicated. **c** UCSC browser screenshot of the microRNAs located on the 14q32 region. Top shows schematic representation of chromosome 14, with the 14q32 locus indicated as a red rectangle. Bottom shows the relative positions of the 39 microRNAs that are positively associated with *PCDH17* expression (left, the 14q32.2 microRNA cluster; right, the 14q32.31 microRNA cluster)

To extend the observation that clinical features differ between AML samples with low versus high *PCDH17* expression, we first compared the transcriptomes of low *PCDH17* expressed group with those of high expression. This comparison yielded 739 differentially expressed genes (FDR < 0.05, |log2 FC| > 1.5; Fig. 8a; Additional file 5), in which 584 genes were positively correlated with *PCDH17* expression levels, and 153 were negatively correlated.

Among the positively associated genes, the *PCDH17* gene itself was ranked as the highest, as expected; other members of the cadherin superfamily, the *HOXC* and *HOXD* gene family, and the collagen family were also identified within this signature. Importantly, several tumor suppressors were found. Among them were negative regulators of epithelial-mesenchymal transition (EMT)—*SLIT2*, *ID4*, and *CDH1*—all three genes were known to be regulated by methylation- and *EZH2*-mediated repression in cancers, and the Wnt antagonists, such as *DKK1*, *WNT5A*, and *SOX17*. One of the most differentially expressed genes was *SPARCL1*, whose repression has been reported as a negative prognostic indicator in a number of cancers [36, 37]. *SPARCL1* is best known for its role as an inhibitor of metastatic-invasion in solid tumors [38]; whether this gene has an antileukemic effect, however, remains to be identified.

As distinct from the tumor suppressive signatures that were positively associated with *PCDH17* expression, those negatively correlated with *PCDH17* expression consists mainly of oncogenes. Among them were known cancer/testis antigen genes (*PNMA5*, *SPATA22*, *ROR1*, and *CT45A6*) and some new candidates (*PAX2*, *HES4*, *PEG10*, *NTN4*, *PDE10A*, and *POSTN*), these genes could be useful tumor markers and potential therapeutic targets. Another noteworthy feature was an enrichment of EMT-inducing factors, including *MMP7*, *BIRC7*, *GLI2*, *PROX1*, and *FGF10*. Three of them—*MMP7*, *BIRC7*, and *PROX1*—were known as transcriptional targets of Wnt/β-catenin signaling [39–41]. *Gli2*, a transcription factor of the Hedgehog pathway, has recently been reported as a negative prognostic factor in AML patients, and its expression was significantly correlated with *FLT3* mutation status [42].

We also derived a *PCDH17* associated microRNA expression signature comprising 69 microRNAs (FDR < 0.05, |log2 FC| > 1.5; Fig. 8b; Additional file 6). Of the 69 microRNAs, 56 (fifty-six) microRNAs were down-regulated and 13 up-regulated in patients with low *PCDH17* expression. To our surprise, more than two-thirds (n = 39) of the microRNAs in the former group were encoded within a microRNA cluster at 14q32 (Fig. 8c). This microRNA cluster, originally described as a human imprinted locus, were frequently deleted or epigenetically silenced in various cancers [43, 44], highlighting its tumor suppressive roles. Specifically, reduced expression of three microRNAs in this cluster (*miR*-*381*, *miR*-*485*, and *miR*-*495*) was associated with multidrug resistance in leukemia [45], and *miR*-*495* has been reported as a tumor suppressor in MLL-rearranged AML [46]. We also identified three microRNAs embedded in the *HOX* cluster (*miR*-*10b*, *miR*-*196a*-*1* and *miR*-*196a*-*2*): while *miR*-*10b* was shown to be negatively correlated with *BAALC* expression in AML [47], *miR*-*196a* was reported as a negative *ERG* regulator [48]. In addition, EMT-related microRNAs were noted, including *miR*-*218*-*2*, which was frequently repressed and co-methylated with its host gene *SLIT3* [49]; *miR*-*214*, which targets *EZH2*, *β*-*catenin*, and *BIRC7* [50]—all were genes implicated in cancer metastasis and invasion; *miR*-*382*, which targets *ROR1* [51]. It is worth noting that *SLIT3*, *BIRC7*, and *ROR1* were all among the aforementioned gene expression signatures. As for those negatively associated with *PCDH17*, only 3 of the 8 genes have previously been associated with carcinogenesis: *miR*-*1269* was shown to mediate tumor metastasis by targeting *HOXD10* in colorectal cancer [52]; the two other microRNAs (*miR*-*508* and *miR*-*135a*-*1*) were mainly implicated in drug resistance.

### GSEA analyses for PCDH17 expression associated transcriptomes

**Fig. 9:**
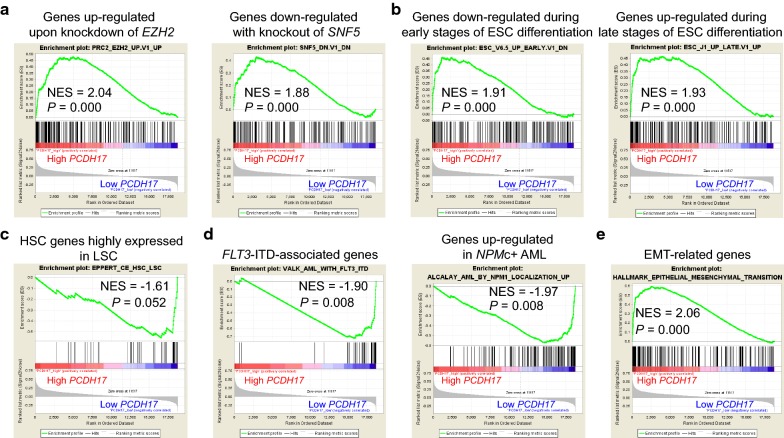
*PCDH17* expression is associated with distinct gene signatures in AML. **a** GSEA plots show enrichment of *EZH2* and *SNF5* gene sets in *PCDH17* high expressers versus low expressers. **b** GSEA comparison of *PCDH17* high expressers versus low expressers shows enrichment of genes down-regulated during early stages of ESC differentiation and genes up-regulated during late stages of ESC differentiation. **c** GSEA plots show up-regulation of AML HSC/LSC signature genes in *PCDH17* low expressers versus high expressers. **d** GSEA plots show enrichment of *FLT3*-ITD and *NPM1* gene sets in *PCDH17* low expressers versus high expressers. **e** GSEA plot shows enrichment of EMT-related signature in *PCDH17* high expressers versus low expressers. The normalized enrichment scores (NES) and *P* values are indicated in each plot. ESC, embryonic stem cell

Considering the above differential analysis might lose some genes of potential biological meanings, we performed a gene set enrichment analysis (GSEA) by comparing transcriptomes of low *PCDH17* expressers with that of high *PCDH17* expressers. Our results confirmed and extended the above findings: (1) high *PCDH17* expressers showed significant enrichment of genes up-regulated upon knockdown of *EZH2* and genes down-regulated with depletion of *SNF5*, an established antagonist gene of *EZH2* (Fig. 9a). It was found that *EZH2*, a component of the Polycomb repressive complex 2 (PRC2), could predispose certain TSGs to cancer-associated promoter hypermethylation during the early stages of stem cell development [53], consistent with our observation that *PCDH17* was significantly hypermethylated in LSCs; (2) transcriptomes of high *PCDH17* expressers are enriched in genes down-regulated during early differentiation of embryonic stem cells (ESCs) and those up-regulated in late stage (Fig. 9b), which agreed favorably with the dynamic expression pattern of *PCDH17* during hematopoiesis; (3) low *PCDH17* expressers tend to express HSC genes that are highly expressed in LSC (Fig. 9c), indicating that the gene itself might be down-regulated in LSC; (4) two earlier gene sets previously correlated with *FLT3*-ITD and *NPM1* mutation were found enriched in the low *PCDH17* signature (Fig. 9d); and (5) as expected, *PCDH17*-associated genes were significantly correlated with the EMT gene signature (Fig. 9e).

Together, these results further demonstrate that low *PCDH17* expression is associated with distinct molecular features in AML.

## Discussion

Our results showed that *PCDH17* was significantly down-regulated in the bulk AML as well as in the LSC populations, as compared to their normal counterparts. This repressed expression, mediated mainly by DNA methylation, was correlated with distinct clinical and biological features. Most importantly, we found *PCDH17* repression to be an independent prognostic factor for shorter OS and DFS in AML; it could add additional prognostic value by stratifying molecularly defined patients into more homogeneous groups.

Although reduced *PCDH17* expression has been reported in a number of tumor types [19], it has, thus far, not been demonstrated in the context of AML. Our study shows that *PCDH17* was repressed in AML and, in particular, in AML stem cells (LSCs). This repression was observed in four microarray profiling datasets of AML and confirmed by RT-qPCR, suggesting down-regulation of *PCDH17* expression as a common alteration in AML. We also tracked *PCDH17* expression during differentiation of major hematopoietic lineages. Despite the well-established role of *PCDH17* in neural development, its involvement in hematopoiesis has, to our knowledge, been rarely documented. Our analysis shows that *PCDH17* transcript levels are highest in the HSC and decrease sharply as HSC differentiate into multipotent progenitor (MPP). This observation was consistent with the findings that adhesion molecules tend to be up-regulated in HSCs and down-regulated upon early commitment [54, 55]. It is possible that high levels of *PCDH17* may facilitate retention of HSCs in the stem cell niche, whereas decreased *PCDH17* expression might result in destabilized cell–matrix interactions, thereby allowing HSCs to mobilize and differentiate. In pathological settings, however, *PCDH17* inactivation might confer upon HSCs/pre-LSCs an enhanced proliferation activity, which cooperates with other phenotypic changes, eventually leading to fully transformed LSCs. Interestingly, we found *PCDH17* expression was increased in the mature cell populations, possibly because *PCDH17* may participate in the homing process of peripheral blood leukocyte.

Several prognostically relevant LSC gene signatures have been reported thus far [10–13]; these signatures consist of genes that are highly expressed not only in LSC but also in HSC, leaving the prognostic value of tumor suppressor genes in LSC largely undetermined. Given that *PCDH17* expression was significantly repressed in LSC, we sought further to investigate its clinical implications. Our results showed that decreased *PCDH17* expression was associated with female sex, higher median WBC counts, as well as higher median PB and BM blast counts, indicative of a high proliferative potential of *PCDH17* repressed leukemia cells. With respect to the prognostic relevance, we found low *PCDH17* expression predicted inferior OS and DFS in the whole TCGA cohort as well as in the subgroup of cytogenetically normal patients; the predictive value for OS was further validated in three independent cohorts. In addition, multivariate analysis demonstrated that *PCDH17* expression was an independent prognostic factor for worse OS and DFS. In the subset of patients with CN-AML, however, *PCDH17* expression was only independently associated with poorer OS, but not with poorer DFS.

Interestingly, we found patients with low *PCDH17* expression harboring *FLT3*-ITD and *NPM1* mutations (the two most common mutations in CN-AML) more often than those with *PCDH17* high expression; GSEA analysis further confirmed this observation. In contrast, low *PCDH17* expression was found to be negatively associated with *TP53* mutations (a mutation occurred frequently in AML with complex aberrant karyotype). These results indirectly reflect the fact that *FLT3*-ITD mutation was often detected together with *NPM1* mutation, whereas *TP53* mutation never or rarely co-occurred with the former two mutations [3, 56]. They also indicate a possible pathogenic link between these mutations and *PCDH17* alteration. It should be noted that, however, these mutations, which themselves have well-recognized prognostic impacts, are potential confounding factors for outcome prediction. We therefore analyzed the prognostic value of *PCDH17* within the mutationally defined subgroups. Low *PCDH17* expression still negatively impacts survival in the *FLT3*-ITD wild-type, *NPM1* mutated, and *TP53* absent patient subsets, suggesting that *PCDH17* expression status could further refine the molecular classification of subsets of AML patients with or without specific mutations.

Finally, we derived *PCDH17*-associated gene/microRNA expression signatures in AML. One prominent feature was the identification of several EMT-related genes, including E-cadherin repressors (i.e., *Gli2*, *CNTN1*, and *CTNND2*), epithelial markers (*CDH1*), matrix metalloproteinases (*MMP7*), and type IV collagens (i.e., *COL4A1* and *COL4A2*). Notably, three EMT-related genes from the signatures (*CDH1*, *BIRC7*, and *Gli2*) were reported by us and others to have profound prognostic significances in AML patients [42, 57, 58]. It was somewhat surprising, however, that EMT modulators could have such implications in leukemia, which, unlike carcinomas, is already endowed with metastatic potential. Recent studies have started to shed some light on this puzzle. For example, *ZEB2*, a classic EMT regulator, has been shown to regulate early hematopoiesis [59]; its depletion caused aberrant differentiation and reduced proliferation of AML cells, indicating cell context-dependent activities of EMT regulators [60]. Moreover, using an MLL-AF9-induced murine model, Stavropoulou et al. [61] have identified an EMT-like gene signature that was linked to short latency, high aggressiveness, and poor prognosis of AML. This might be explained by the proinvasive and stem-like properties associated with deregulated EMT-like transcriptomes. Future research should further investigate their distinct roles in the context of hematological malignancies.

Another interesting finding was the deregulated expression of Wnt signaling pathway genes, including Wnt antagonists (i.e., *DKK1*, *WNT5A*, and *SOX17*) and Wnt targets (i.e., *MMP7*, *BIRC7*, and *PROX1*). Indeed, *PCDH17* was shown to be an inhibitor of the Wnt/β-catenin signaling in breast cancer [30]; the same might also hold true in AML. Thus, it is possible that repressed *PCDH17* contributes Wnt pathway activation and enhanced proliferation of leukemic cells, which is consistent with our observation of higher WBC counts and percentages of BM and PB blasts in patients with low *PCDH17* expression. In addition to *PCDH17*, our earlier studies have established the prognostic significances of some other Wnt antagonists in AML, such as *SOX17*, *NKD2*, and *SFRP* genes [31–33], highlighting the importance of deregulated Wnt signaling in leukemogenesis.

For microRNAs co-expressed with *PCDH17*, some of them were previously reported to be negatively associated with the unfavorable prognostic factors in AML-that is, *MDR1* (i.e., *miR*-*381*, *miR*-*485*, and *miR*-*495*) [45], *BAALC* (*miR*-*10b*) [47], and *ERG* (*miR*-*196a*) [48], further supporting the negative prognostic significance of deregulated *PCDH17*.

## Conclusions

In summary, low *PCDH17* expression is an independent, poor prognostic factor in AML and has additional prognostic value in stratifying molecularly defined subgroups. Furthermore, low *PCDH17* expressers are characterized by a gene-expression signature that reflects the deregulations of EMT- and Wnt pathway-related genes. It is possible that these deregulations contribute to the adverse outcome observed in low *PCDH17* patients. Future studies are warranted to confirm this hypothesis.

## Additional files

**Additional file 1.** This file contains Additional Figures (Figures S1–S2) and Additional Tables (Tables S1–S3). It also includes Additional Methods information and R code for regenerating the LSC17 score.

**Additional file 2.** List of 443 down-regulated probes/genes in LSCs versus normal HSCs.

**Additional file 3.** List of 863 hypermethylated CpGs in LSCs versus normal HSCs.

**Additional file 4.** Methylation levels of *PCDH17* as measured by targeted bisulfite sequencing.

**Additional file 5.** Differentially expressed genes between AML patients with low and high *PCDH17* expression.

**Additional file 6.** Differentially expressed microRNAs between AML patients with low and high *PCDH17* expression.

## Abbreviations

LSC: leukemia stem cell
AML: acute myeloid leukemia
TSG: tumor suppressor gene
HSC: hematopoietic stem cell
TCGA: The Cancer Genome Atlas
PCDH17: protocadherin17
BM: bone marrow
PB: peripheral blood
OS: overall survival
ESCC: esophageal squamous cell carcinoma
RT-qPCR: real-time quantitative PCR
FACS: fluorescence-activated cell sorting
FLASH: fast length adjustment of short reads
PCA: principal component analysis
s: 5-aza-2′-deoxycytidine
RQ-MSP: real-time methylation-specific polymerase chain reaction
DFS: disease-free survival
FDR: false discovery rate
GSEA: gene set enrichment analysis
MNC: mononuclear cell
PMN: polymorphonuclear cell
WBC: white blood cell
CN: cytogenetically normal
allo-HSCT: allogeneic stem-cell transplantation
CR: complete remission
EMT: epithelial-mesenchymal transition
PRC2: polycomb repressive complex 2
ESC: embryonic stem cell
MPP: multipotent progenitor
