# Supplementary material for: Reduced protocadherin17 expression in leukemia stem cells: the clinical and biological effect in acute myeloid leukemia
Source: J Transl Med. 2019 Mar 29;17:102. doi: 10.1186/s12967-019-1851-1 (PMC6440111; doi:10.1186/s12967-019-1851-1)
Supplement: Supplementary file 1 — Additional file 1. This file contains Additional Figures (Figures S1–S2) and Additional Tables (Tables S1–S3). It also includes Additional Methods information and R code for regenerating the LSC17 score. [file 12967_2019_1851_MOESM1_ESM.docx]

**Reduced protocadherin17 expression in leukemia stem cells: the clinical and biological effect in acute myeloid leukemia**

**This file contains Additional Figures (Figures S1–S2) and Additional Tables (Tables S1–S3). It also includes Additional Methods information and R code for regenerating the LSC17 score.**

**Table of Contents**

[Additional Figures 3](#_Toc1071759)

[Additional Tables 5](#_Toc1071760)

[Additional Methods 8](#_Toc1071761)

[R script for recalculating the LSC17 score 13](#_Toc1071762)

# Additional Figures

**
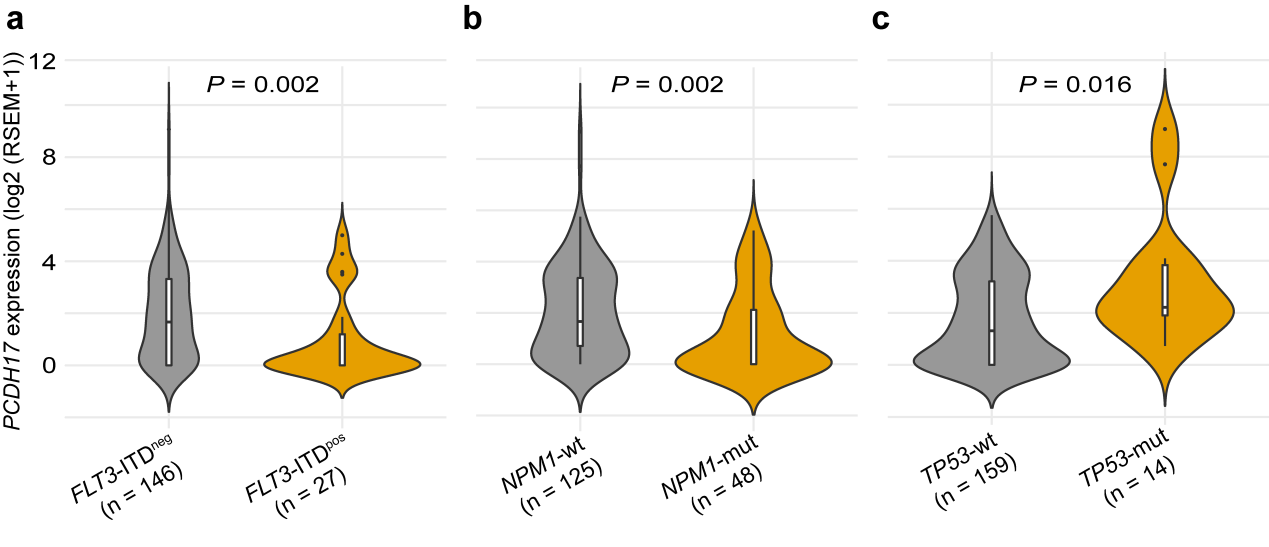
**

**Figure S1.** Comparisons of *PCDH17* expression between the AML groups with or without certain mutations. (**a**) *FLT3*-ITD negative (*FLT3*-ITD^neg^) and *FLT3*-ITD positive (*FLT3*-ITD^pos^) AML patients, (**b**) *NPM1* wild-type (*NPM1*-wt) and *NPM1* mutated (*NPM1*-mut) AML patients, (**c**) *TP53* wild-type (*TP53*-wt) and *TP53* mutated (*TP53*-mut) AML patients. *PCDH17* expression was significantly down-regulated in the *FLT3*-ITD positive, *NPM1* mutated, and *TP53* wild-type groups as compared with their genetically defined counterparts. RSEM normalized count of *PCDH17* (from TCGA data set) were log2 transformed (log2 (RSEM+1)) to generate the violin plots. The *P* values were calculated using the Wilcoxon rank-sum test.

**
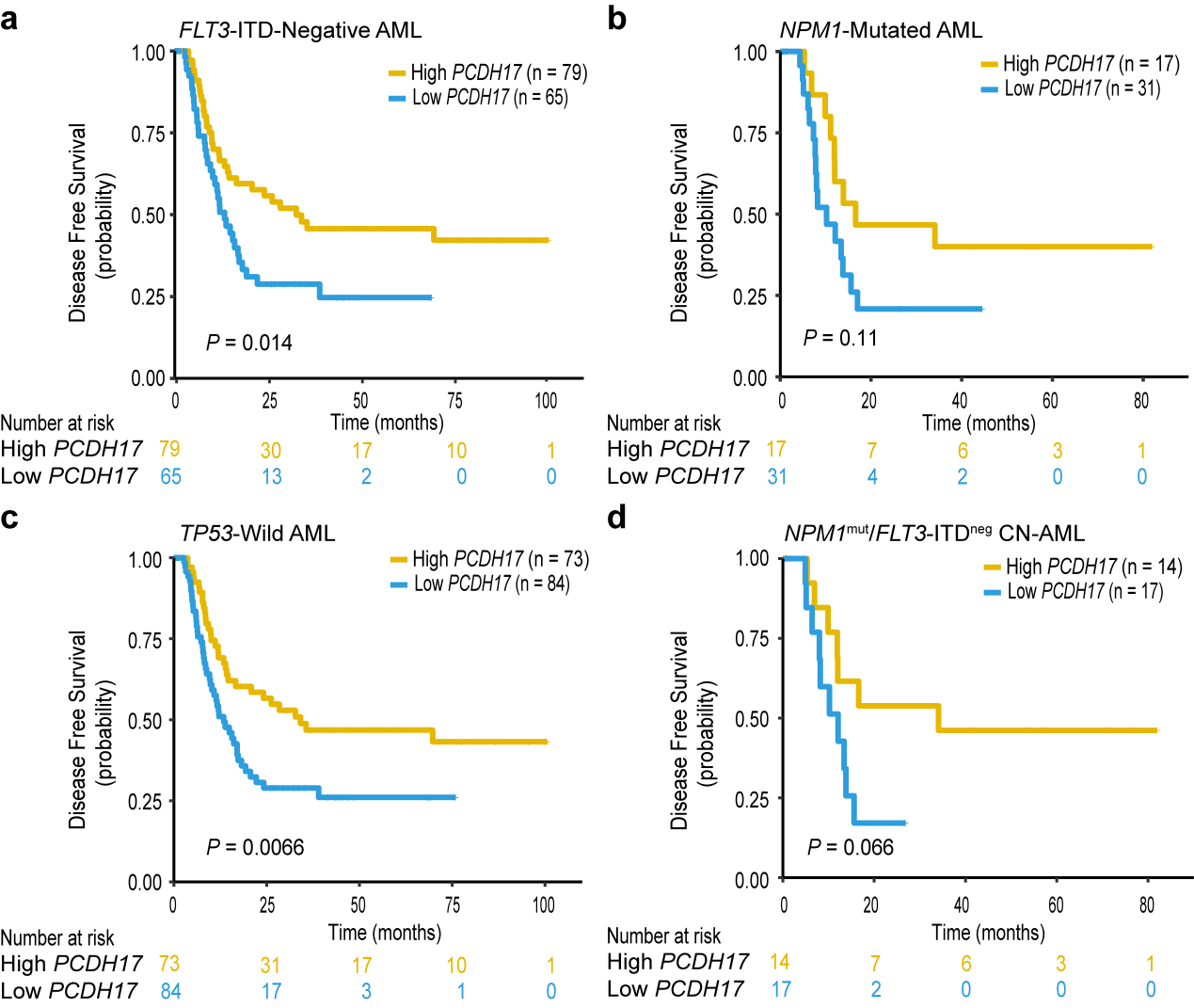
**

**Figure S2.** Reduced *PCDH17* expression is associated with worse DFS in the genetically defined subgroups of AML patients, as indicated. Low *PCDH17* expression predicts reduced DFS only in the *FLT3*-ITD absent (**a**) and *TP53* wild-type (**c**) AML subgroups.

# Additional Tables

| **Table S1.** **Details of public datasets used in this study** | | | | | |
| --- | --- | --- | --- | --- | --- |
| Dataset | Data type | Platform(s) | Sample type | No. of samples | Reference |
| TCGA | RNA-seq | Illumina HiSeq 2000 | AML | 173 | Ley, TJ, et al (2013) |
| TCGA | miRNA-seq | Illumina Genome Analyzer | AML | 188 | Ley, TJ, et al (2013) |
| GSE12417 | microarray | Affymetrix U133 Plus 2.0  Affymetrix U133B | AML | 242 | Metzeler,  KH, et al (2008) |
| GSE24006 | microarray | Affymetrix U133 Plus 2.0 | AML, NBM | 54 | Gentles, AJ, et al (2010) |
| GSE30029 | microarray | Illumina HumanHT-12 V3.0 | AML, NBM | 77 | de Jonge, HJ, et al (2011) |
| GSE42519 | microarray | Affymetrix U133 Plus 2.0 | NBM | 34 | Rapin, N, et al (2014) |
| GSE63270 | microarray | Affymetrix U133 Plus 2.0 | AML, NBM | 104 | Jung, N, et al (2015) |
| GSE63409 | DNA methylation array | Illumina HumanMethylation450 | AML, NBM | 74 | Jung, N, et al (2015) |

| **Table S2.** **Primer sequences used in this study** | | |
| --- | --- | --- |
| Primers | Primer sequence (5’ to 3’) | Predicted product size (bp) |
| RQ-PCR primers | | |
| *PCDH17*-forword | GAGCAACAGAATCACCATAC | 148 |
| *PCDH17*-reverse | GCAGTTCCACCAAGTAGG |  |
| RQ-MSP primers | | |
| *PCDH17*-M-forword | TTTCGTTGGGTATAGTGGTTATC | 149 |
| *PCDH17*-M- reverse | CACACGAATTAAACCGATCTTA |  |
| *PCDH17*-U-forword | GTTTTGTTGGGTATAGTGGTTATT | 151 |
| *PCDH17*-U-reverse | TCACACAAATTAAACCAATCTTA |  |
| Targeted bisulfite sequencing primers | | |
| *PCDH17*-forword | TGTAGTTTGGGTTTTYGTTTGTAGAG | 233 |
| *PCDH17*-reverse | CTCTACCTTAATCATACAAATCTCCTTATC |  |

| **Table S3. Univariate analysis of *PCDH17* expression for overall survival and disease-free survival in the TCGA cohort** | | | | |
| --- | --- | --- | --- | --- |
| **Variables** | **Overall survival** | | **Disease-free survival** | |
|  | **Hazard Ratio (95% CI)** | ***P*** | **Hazard Ratio (95% CI)** | ***P*** |
| **Full cohort** | **(n = 173)** | | **(n = 171)** | |
| *PCDH17*^a^ | 1.55 (1.07-2.25) | 0.021 | 1.73 (1.11-2.68) | 0.015 |
| Age^b^ | 3.14 (2.15-4.57) | < 0.0001 | 1.39 (0.89-2.18) | 0.15 |
| WBC count^c^ | 1.21 (0.84-1.76) | 0.31 | 2.03 (1.31-3.14) | 0.0014 |
| Cytogenetic risk^d^ | 2.02 (1.50-2.71) | < 0.0001 | 1.61 (1.14-2.26) | 0.0069 |
| *FLT3*-ITD^e^ | 1 (0.60-1.68) | 0.99 | 1.28 (0.72-2.28) | 0.40 |
| *NPM1*^f^ | 1.16 (0.77-1.73) | 0.48 | 1.31 (0.82-2.09) | 0.27 |
| *CEBPA*^f^ | 0.93 (0.47-1.83) | 0.83 | 1.40 (0.67-2.91) | 0.37 |
| *TP53*^f^ | 4.11 (2.30-7.35) | < 0.0001 | 2.36 (0.94-5.89) | 0.067 |
| *DNMT3A*^f^ | 1.57 (1.04-2.38) | 0.032 | 1.57 (0.96-2.56) | 0.075 |
| *RUNX1*^f^ | 1.84 (1.03-3.30) | 0.04 | 1.35 (0.62-2.94) | 0.45 |
| *IDH1*^f^ | 0.71 (0.36-1.40) | 0.33 | 0.65 (0.28-1.49) | 0.31 |
| *IDH2*^f^ | 1.03 (0.57-1.88) | 0.92 | 0.95 (0.48-1.91) | 0.89 |
| **CN-AML** | **(n = 80)** | | **(n = 80)** | |
| *PCDH17*^a^ | 2.22(1.26-3.92) | 0.0059 | 2.02 (1.12-3.65) | 0.019 |
| Age^b^ | 2.24 (1.31-3.84) | 0.0034 | 1.70 (0.96-3.02) | 0.069 |
| WBC count^c^ | 1.57 (0.92-2.70) | 0.099 | 1.68 (0.95-2.98) | 0.074 |
| *FLT3*-ITD^e^ | 1.19(0.62-2.27) | 0.61 | 1.55 (0.80-2.98) | 0.2 |
| *NPM1*^f^ | 1.06 (0.62-1.81) | 0.82 | 0.90 (0.51-1.58) | 0.71 |
| *CEBPA*^f^ | 1.01 (0.43-2.37) | 0.98 | 1.29 (0.55-3.05) | 0.56 |
| *TP53*^f^ | 2.61 (0.35-19.24) | 0.35 | 5.23 (0.68-40.21) | 0.11 |
| *DNMT3A*^f^ | 1.78 (1.03-3.08) | 0.041 | 2.07 (1.15-3.73) | 0.015 |
| *RUNX1*^f^ | 1.27 (0.50-3.21) | 0.62 | 1.57 (0.62-3.99) | 0.34 |
| *IDH1*^f^ | 0.44 (0.16-1.22) | 0.11 | 0.48 (0.17-1.35) | 0.16 |
| *IDH2*^f^ | 1 (0.45-2.21) | 0.99 | 1.06 (0.47-2.38) | 0.88 |

Abbreviations: TCGA, The Cancer Genome Atlas; CI, confidence interval; WBC, white blood cells; CN-AML, cytogenetically normal AML; ITD, internal tandem duplication.

NOTE: Hazard Ratio > 1 or Hazard Ratio < 1 indicate a higher or lower risk. P ≤ 0.20 were highlighted.

^a^Low vs high expression.

^b^> 60 vs ≤ 60 years.

^c^≥ 30 vs < 30 ×10^9^/L.

^d^Adverse vs intermediate vs favorable.

^e^Present vs absent.

^f^Mutated vs wild type.

# Additional Methods

*Patient characteristics of the published cohorts*

The discovery cohort was derived from The Cancer Genome Atlas (TCGA) dataset, including 200 de novo AML patients diagnosed between 2001 to 2010 (median age, 57 years; range,18-88 years) [[1](#_ENREF_1)]. All patients were treated in accordance with NCCN guidelines ([www.nccn.org](http://www.nccn.org)). 70 out of 158 patients with intermediate or unfavorable cytogenetics underwent allogeneic stem cell transplant, and 67 of them have RNA-sequencing data. Survival analysis was based on 173 cases for which expression and survival data were available. The clinical endpoints analyzed included overall and event-free survival (OS and DFS). This study was approved by the Washington University Human Studies Committee with written informed consent obtained from all patients.

We also evaluated the prognostic value of *PCDH17* expression in two large cytogenetically normal AML cohorts (median age: 47 years; range: 17–59 years; cohort1, n = 79; cohort2, n = 163) [[2](#_ENREF_2)]. All but 17 patients received intensive double-induction and consolidation chemotherapy according to the AMLCG-1999 protocol. Gene expression and outcome data (OS only) were obtained from Gene Expression Omnibus (http://www.ncbi.nlm.nih.gov/geo/, accession number GSE12417. The study was approved by the local institutional review boards, and informed consent was obtained from all patients in accordance with the Declaration of Helsinki.

The clinical analyses of *PCDH17*, including correlation analysis of clinical parameters, survival analysis in the context of other molecular markers, were largely based on the TCGA cohort, because extensively annotated clinical data were available for this cohort.

*Analysis of the TCGA data*

**Data information:** For the TCGA cohort, normalized RNAseqV2 data, mutational data, and clinical data were downloaded from cBioPortal for Cancer Genomics (<http://www.cbioportal.org>), while raw read counts of RNA/microRNA sequencing was obtained from Broad Firehose (<http://gdac.broadinstitute.org>). 173 patients with RNA sequencing data were classified by median *PCDH17* expression as “high *PCDH17*” (n = 86) or “low *PCDH17*” (n = 87). Eight frequently mutated genes with known prognostic implications in AML- *FLT3-ITD, NPM1, CEBPA, IDH1, IDH2, RUNX1, DNMT3A,* and *TP53-*were retrieved from the mutational data and included in the clinical analyses.

**Recalculation of the LSC17 score:** To recalculate the LSC17 score [[3](#_ENREF_3)], expression values of the reported 17 genes were obtained from the Z-score-transformed TCGA data. After scaling and centering, the Z-scores were weighted by the regression coefficients to generate the risk score. A median risk score was used to divide patients into high- and low-risk groups. For the microarray data (GSE12417), probe set with the highest mean expression was selected to represent each gene; other procedures were as described above.

**Differential expression analysis:** Gene/microRNA expression differences between high and low *PCDH17* groups were calculated using the raw read counts with the R/Bioconductor package “edgeR” [[4](#_ENREF_4)]. Genes/microRNAs with Benjamini–Hochberg (BH)-adjusted p-values < 0.05 were considered differentially expressed. Results of the differential analyses were represented as volcano plot and heatmaps (Fig. 8), and the heatmaps were visualized with “pheatmap” package.

**Gene Set Enrichment Analysis (GSEA):** GSEA was performed on the TCGA dataset using GSEA v3.0 software (<http://www.broad.mit.edu/gsea>) [[5](#_ENREF_5), [6](#_ENREF_6)]. Statistical significance of GSEA results was determined by 1,000 gene set permutations, with signal-to-noise gene ranking. All the gene sets used in this study were obtained from GSEA MSigDB website (http://software.broadinstitute.org/gsea/msigdb/index.jsp), and the gene sets were considered to be significantly enriched at a false discovery rate < 0.25 and normalized p-value < 0.05. Three categories of gene sets were used in this study: C2, curated gene sets containing genes coregulated in response to specific perturbations; C6, oncogenic gene sets that reflect genes commonly dysregulated in cancer; and H, hallmark gene sets which represent well-defined biological states or processes.

*Analysis of the GEO data*

**Data information:** A total of 6 microarray data sets (including one DNA methylation array) from GEO database were used in this study. The accession number and platform information for each data set were described in Table S1. All gene expression cohorts were individually normalized as described in the original studies [[2](#_ENREF_2), [7-10](#_ENREF_7)]. In case of multiple probes per gene, *PCDH17* expression was determined using the 205656_at probe for HG-U133 Plus 2.0 platform, and 227289_at for HG-U133B platform. To generate the box plots in Fig.1 and Supplementary Fig. 1, the expression values of *PCDH17* were log2-transformed in all data sets, except for that of GSE30029, which contains negative values in the original data.

**Differential expression/methylation analyses:** To identify candidate tumor suppressor genes in leukemia stem cells (LSCs), we utilized two data sets (GSE63270 and GSE63409) described by Jung N et al.[[10](#_ENREF_10)]. Data were analyzed as follows: Expression/methylation data of LSC and HSC samples were extracted from the two datasets. Then, Differential expression/methylation analyses were performed using the R package “limma” [[11](#_ENREF_11)]. To find significantly differentially methylated genes, only CpG probes located in gene promoter regions (CpG sites located within 200 bp or 1,500 bp of the transcription start site (TSS) and in the 5′untranslated region and exon 1) were selected.

**References**

1. Ley TJ, Miller C, Ding L, Raphael BJ, Mungall AJ, Robertson A, Hoadley K, Triche TJ, Jr., Laird PW, Baty JD, et al: Genomic and epigenomic landscapes of adult de novo acute myeloid leukemia**.** *N Engl J Med* 2013; 368**:**2059-2074.

2. Metzeler KH, Hummel M, Bloomfield CD, Spiekermann K, Braess J, Sauerland MC, Heinecke A, Radmacher M, Marcucci G, Whitman SP, et al: An 86-probe-set gene-expression signature predicts survival in cytogenetically normal acute myeloid leukemia**.** *Blood* 2008; 112**:**4193-4201.

3. Ng SW, Mitchell A, Kennedy JA, Chen WC, McLeod J, Ibrahimova N, Arruda A, Popescu A, Gupta V, Schimmer AD, et al: A 17-gene stemness score for rapid determination of risk in acute leukaemia**.** *Nature* 2016; 540**:**433-437.

4. Robinson MD, McCarthy DJ, Smyth GK: edgeR: a Bioconductor package for differential expression analysis of digital gene expression data**.** *Bioinformatics* 2010; 26**:**139-140.

5. Subramanian A, Tamayo P, Mootha VK, Mukherjee S, Ebert BL, Gillette MA, Paulovich A, Pomeroy SL, Golub TR, Lander ES, Mesirov JP: Gene set enrichment analysis: a knowledge-based approach for interpreting genome-wide expression profiles**.** *Proc Natl Acad Sci U S A* 2005; 102**:**15545-15550.

6. Mootha VK, Lindgren CM, Eriksson KF, Subramanian A, Sihag S, Lehar J, Puigserver P, Carlsson E, Ridderstrale M, Laurila E, et al: PGC-1alpha-responsive genes involved in oxidative phosphorylation are coordinately downregulated in human diabetes**.** *Nat Genet* 2003; 34**:**267-273.

7. Gentles AJ, Plevritis SK, Majeti R, Alizadeh AA: Association of a leukemic stem cell gene expression signature with clinical outcomes in acute myeloid leukemia**.** *Jama* 2010; 304**:**2706-2715.

8. de Jonge HJ, Woolthuis CM, Vos AZ, Mulder A, van den Berg E, Kluin PM, van der Weide K, de Bont ES, Huls G, Vellenga E, Schuringa JJ: Gene expression profiling in the leukemic stem cell-enriched CD34+ fraction identifies target genes that predict prognosis in normal karyotype AML**.** *Leukemia* 2011; 25**:**1825-1833.

9. Rapin N, Bagger FO, Jendholm J, Mora-Jensen H, Krogh A, Kohlmann A, Thiede C, Borregaard N, Bullinger L, Winther O, et al: Comparing cancer vs normal gene expression profiles identifies new disease entities and common transcriptional programs in AML patients**.** *Blood* 2014; 123**:**894-904.

10. Jung N, Dai B, Gentles AJ, Majeti R, Feinberg AP: An LSC epigenetic signature is largely mutation independent and implicates the HOXA cluster in AML pathogenesis**.** *Nat Commun* 2015; 6**:**8489.

11. Ritchie ME, Phipson B, Wu D, Hu Y, Law CW, Shi W, Smyth GK: limma powers differential expression analyses for RNA-sequencing and microarray studies**.** *Nucleic Acids Res* 2015; 43**:**e47.

# R script for recalculating the LSC17 score

R script for recalculating the LSC17 score

#### TCGA Cohort

#### data files can be downloaded from cBioPortal for Cancer Genomics

#### <http://www.cbioportal.org>

# load packages required for analysis

library(xlsx)

library(dplyr)

library(survival)

## Prepare Expression Data

# prepare the Z-score transformed data

data_file <- file.path(

"~/aml_tcga_pub /data_RNA_Seq_v2_mRNA_median_Zscores.txt"

)

expression <- read.table(data_file, sep = "\t",

header = TRUE, row.names = 1, as.is = TRUE)[,-1]

# transpose rows (genes) and columns (samples)

expression <- data.frame(t(expression))

# select the 17 genes in the LSC17 signature

genes <- data.frame(

expression$DNMT3B, expression$ZBTB46, expression$NYNRIN,

expression$ARHGAP22, expression$LAPTM4B, expression$MMRN1,

expression$DPYSL3, expression$KIAA0125, expression$CDK6,

expression$CPXM1, expression$SOCS2, expression$SMIM24,

expression$EMP1, expression$NGFRAP1, expression$CD34,

expression$AKR1C3, expression$GPR56

)

# normalize the data (mean-centered and scaled according to the standard deviation)

genes <- data.frame(scale(genes))

# insert a column with sample ID

genes$SAMPLE_ID <- rownames(expression)

genes$SAMPLE_ID <- substr(genes $SAMPLE_ID, 9, 12)

# average over those with multiple measurements

genes_ag <- aggregate(.~SAMPLE_ID, FUN=mean, data= genes)

## Prepare Survival Data

# read the manually curated clinical data

clinical <- read.xlsx("~/data_clinical.xlsx", 1)

# select the survival data

OS <- select(clinical, SAMPLE_ID, OS_MONTHS, OS_STATUS)

OS$SAMPLE_ID <- substr(OS$SAMPLE_ID, 9, 12)

# create binary variable for OS status

OS$fdelva <- ifelse(OS$OS_STATUS == "DECEASED", 1, 0)

OS$fdelva <- as.factor(OS$fdelva)

# merge the expression and survival data

dataM <- merge(OS, genes_ag, by = "SAMPLE_ID")

## Calculate the LSC17 Score

coefs <- c(0.0874, -0.0347, 0.00865, -0.0138, 0.00582, 0.0258, 0.0284, 0.0196, -0.0704, -0.0258, 0.0271, -0.0226, 0.0146, 0.0465, 0.0338, -0.0402, 0.0501)

coefs <- data.frame("coefs" = coefs)

coefs <- as.matrix(coefs$coefs)

risk_score <- as.matrix((dataM[, 5:21] - colMeans(dataM[, 5:21]))) %*% coefs

dataM$risk_score <- risk_score

## Survival Analysis

dataM$risk_cat <- ifelse(dataM$risk_score > median(dataM$risk_score), "high", "low")

dataM$risk_cat <- as.factor(dataM$risk_cat)

survdiff(Surv(OS_MONTHS, fdelva==1) ~ risk_cat, data = dataM)

#### GSE12417 Cohort 1

#### data files can be downloaded from Gene Expression Omnibus

#### <http://www.ncbi.nlm.nih.gov/geo/>, accession number GSE12417

# load packages required for analysis

library(xlsx)

library(hgu133plus2.db)

library(annotate)

library(survival)

## Prepare Expression Data

# prepare the normalized microarray data

data_file <- file.path("~/ GSE12417-GPL570_series_matrix.txt")

expression <- as.matrix(read.table(data_file, sep = "\t",

header = TRUE, row.names = 1, as.is = TRUE))

## obtain the probe sets of the 17 genes in the LSC17 signature

xx <- as.list(hgu133plus2ALIAS2PROBE)

genes <- c("DNMT3B", "ZBTB46", "NYNRIN", "ARHGAP22", "LAPTM4B", "MMRN1", "DPYSL3", "KIAA0125", "CDK6", "CPXM1", "SOCS2", "SMIM24", "EMP1", "NGFRAP1", "CD34", "AKR1C3", "GPR56")

list <- xx[match(genes,names(xx))]

probes <- unlist(list,use.names=FALSE)

# subset the 43 probe sets from the gene expression matrix

probesets <- as.data.frame(expression[rownames(expression) %in% probes, ])

# reannotate these probe sets into gene symbles

sym <- getSYMBOL(rownames(probesets), "hgu133plus2.db")

genes <- data.frame(sym, probesets)

# select the probeset with the highest average expression to represent each gene

genes$mean <- apply(probesets, 1, mean)

genes <- genes[order(genes$sym, genes$mean, decreasing = TRUE),]

genes <- genes[!duplicated(genes$sym),]

# normalize the data (mean-centered and scaled according to the standard deviation)

rownames(genes) <- genes$sym

genes$sym <- NULL

genes$mean <- NULL

genes <- data.frame(t(genes))

genes <- data.frame(scale(genes))

# insert a column with sample ID

genes$SAMPLE_ID <- rownames(genes)

## Prepare Survival Data

# read the manually curated clinical data including three columns (SAMPLE_ID,

# OS_STATUS, and OS_MONTHS)

OS <- read.xlsx("~/data_clinical.xlsx", 1)

# merge the expression and survival data

dataM <- merge(OS, genes, by = "SAMPLE_ID")

## Calculate the LSC17 Score

coefs <- c(-0.0347, 0.0271, -0.0226, 0.00865, 0.0258, 0.00582, 0.0196, 0.0146, 0.0284, 0.0874, -0.0258, -0.0704, 0.0338, 0.0465, -0.0138, -0.0402, 0.0501)

coefs <- data.frame("coefs" = coefs)

coefs <- as.matrix(coefs$coefs)

risk_score <- as.matrix((dataM[, 4:20] - colMeans(dataM[, 4:20]))) %*% coefs

dataM$risk_score <- risk_score

## Survival Analysis

dataM$risk_cat <- ifelse(dataM$risk_score > median(dataM$risk_score), "high", "low")

dataM$risk_cat <- as.factor(dataM$risk_cat)

survdiff(Surv(OS_MONTHS, OS_STATUS == 1) ~ risk_cat, data = dataM)
